# MIR-21 regulating distribution of intestinal flora through TNF-α promotes progression of ulcerative colitis

**DOI:** 10.5937/jomb0-43320

**Published:** 2024-04-23

**Authors:** Yang Ke, Xueni Liu, Tao Niu, Zhao Qiang, Feng Gao

**Affiliations:** 1 Gansu Provincial Maternity and Child-Care Hospital (Gansu Provincial Central Hospital), Department of Hematology and Oncology, Lanzhou, China; 2 PLA Rocket Force Characteristic Medical Center, Critical Care Medicine, Beijing, China; 3 Gansu Provincial Maternity and Child-Care Hospital (Gansu Provincial Central Hospital), Department of Gastroenterology, Lanzhou, China; 4 Central Committee of the Communist Party of China, Health Service Department of the Guard Bureau of the General Office, Beijing, China

**Keywords:** ulcerative colitis, intestinal flora, miR-21, TNF-α, ulcerozni kolitis, crevna flora, miR-21, TNF-α

## Abstract

**Background:**

To study the changes in intestinal flora in patients with ulcerative colitis (UC), and to explore its correlations with micro ribonucleic acid (miR)-21 and serum tumor necrosis factor-a (TNF-α).

**Methods:**

A total of 150 patients with UC were selected and divided into remission group and seizure group according to the severity of disease. At the same time, 150 healthy people receiving physical examination in the hospital during the same period were selected as control group. The levels of fecal miR-21 and TNF-α in all subjects were determined via reverse transcription-polymerase chain reaction (RT-PCR). The correlation between miR-21 and TNF-α and their associations with the changes in intestinal bacteria in UC were analyzed using Pearson correlation analysis. The risk factors affecting the occurrence of UC were explored via multivariate logistic regression analysis.

## Abstract (sr)

**Uvod:**

Cilj je bio da se prouče promene u crevnoj flori kod pacijenata sa ulceroznim kolitisom (UC) istraže njene korelacije sa mikro ribonukleinskom kiselinom (miR)-21 i serumskog tumor nekroza faktora-a (TNF-α).

**Metode:**

Odabrano je ukupno 150 pacijenata sa UC i podeljeno u grupu remisije i grupu sa napadima prema težini bolesti. Istovremeno, za kontrolnu grupu odabrano je 150 zdravih ljudi koji su u istom periodu bili na bolničkom pregledu. Nivoi fekalnog miR-21 i TNF-α kod svih ispitanika određivani su lančanom reakcijom reverzne transkripcije-polimeraze (RT-PCR). Korelacija između miR-21 i TNF-α i njihova povezanost sa promenama u crevnim bakterijama u UC analizirana je Pirsonovom korelacionom analizom. Faktori rizika koji utiču na pojavu UC istraženi su multi-varijantnom logističkom regresionom analizom.

**Rezultati:**

Nivoi fekalnog miR-21 i TNF-α kod pacijenata sa UC bili su značajno viši od onih u kontrolnoj grupi, a takođe su bili značajno viši u grupi sa napadima nego u grupi sa remisijom. Postojala je pozitivna korelacija između nivoa miR-21 i TNF-α. Broj fekalne crevne flore (Bifidobacterium, Lactobacillus, Enterobacterium i Enterococcus) bio je različit kod pacijenata sa različitim stepenom bolesti. MiR-21 i TNF-α su u negativnoj korelaciji sa sadržajem Bifidobacterium i Lactobacillus, ali u pozitivnoj korelaciji sa sadržajem Enterobacterium i Enterococcus. Prema multivarijantnoj logističkoj regresionoj analizi, miR-21 i TNF-α su bili faktori rizika za napad UC.

**Zaključak:**

MiR-21 promoviše ekspresiju TNF-α i dovodi do promene crevne flore, čime se povećava pojava i razvoj UC.

## Introduction

Ulcerative colitis (UC) is a chronic intestinal disease that arises due to factors such as abnormal immunoregulation and alterations in intestinal microecology. The disease is characterized by symptoms such as abdominal pain, diarrhea, and rectal bleeding [1]. UC is a recurrent condition that is difficult to cure completely. With the progression of the disease, complications such as intestinal perforation and bleeding are likely to occur, significantly impacting the patient’s quality of life [2] [3].

The human body’s intestinal flora is regulated by microorganisms and human genes in a “supraindividual” manner [4]. There are thousands of different types of intestinal bacteria in humans, and their total number can reach 100 trillion, which is 10 times greater than the number of host cells [5]. In recent years, increasing research evidence has suggested that alterations in intestinal flora are closely linked to the occurrence of UC. One study [6] demonstrated that changes in intestinal flora may be the initiating factor for the onset and progression of UC.

Micro ribonucleic acids (miRNAs) are a class of endogenous small RNAs with 20-24 ribonucleotides in length. The regulation of miRNAs on target genes mainly depends on the degree of complementary pairing with the target gene transcript sequence [7]. Takagi et al. [8] performed the miRNA chip analysis and verified 7 miRNAs (let-7a, let-7c, let-7d, let-7g, miR-21, miR-155 and miR-923) via quantitative reverse transcription-polymerase chain reaction (qRT-PCR) based on the screening results. The results showed that compared with those in normal control group, the expression levels of miR-21 and miR-155 in colonic mucosa tissues of active UC patients significantly rise, suggesting that miR-21 and miR-155 may be involved in inflammatory and immune responses of intestinal mucosa.

Tumor necrosis factor-α (TNF-α) is involved in apoptosis and cell proliferation. It not only participates as a pro-inflammatory cytokine in a wide range of human diseases, including inflammatory diseases, but also can lead to tumor development [9] [10]. Recent studies have demonstrated that Saccharo myces cerevisiae can balance the intestinal flora through reducing the level of TNF-α, thereby alleviating UC in mice [11]. In addition, many studies have shown that TNF-α regulated by miRNAs can play an important role in disease progression. For example, miR-21 inhibits TNF-α-induced apoptosis via activating JNK/p38/caspase-3 signaling pathway, thereby promoting the occurrence of acute myocardial infarction in the elderly [12]. However, the associations of miR-21 and TNF-α with UC has not been studied yet. In this study, therefore, the associations of changes in intestinal flora in vivo with miR-21 and TNF-α were mainly analyzed, so as to provide important references for the research on the pathogenesis and clinical treatment of UC.

## Materials and methods

### General data

A total of 150 patients with UC diagnosed and treated in Yantaishan Hospital were selected anddivided into remission group and seizure group according to the severity of disease. Inclusion criteria: 1) patients diagnosed with UC via clinical, laboratory, imaging, endoscopic and histopathologic examinations [13], and 2) the activity of UC was evaluated using the Sutherland score, and the UC activity index >2 indicates seizure stage of UC, or it indicates remission stage of UC. Pregnant women or UC patients who had intestinal infections currently, or took antibiotics, probiotics or yogurt within 2 months were excluded. Another 150 healthy people without taking antibiotics, probiotics and yogurt within 3 months and without gastrointestinal symptoms were enrolled as control group. This study was approved by the ethics committee of Yantaishan Hospital. Signed written informed consents were obtained from all participants before the study.

### Intestinal flora detection

About 1.5 g of fresh feces were collected from the three groups, placed into bottles, diluted with 4.5 mL of diluent, and shaken with a mixer at 300 rpm for 1 min. The samples diluted 10-fold were evenly placed in different bottles, and 10 mL of them were inoculated on the medium and stored at 37°C. The viable count of such beneficial bacteria as Bifidobacterium and Lactobacillus, and such pathogenic bacteria as Enterobacterium and Enterococcus was detected using the logarithmic value of colony forming unit in wet mass per gram of feces (lgCFU/g).

### MRNA extraction

RNAfixer test tubes containing feces samples were centrifuged at 12,000 rpm and 4°C for 10 min. After the liquid was discarded, 1 mL of TRIpure was added into each tube and mixed evenly, and the tubes were placed at room temperature for 10 min. Then 0.2 mL of chloroform was added, and each tube was shaken evenly for 15 d and placed for 5 min, followed by centrifugation at 12,000 rpm and 4°C for 15 min. 0.5 mL of isopropanol was added into new 1.5 mL centrifuge tubes, placed in a refrigerator at -20°C for 1 d, and added with centrifugal supernatant extracted, followed by centrifugation again for 15 min. The next day, the centrifuge tube was taken out, and the precipitate at the bottom was retained, but the liquid was poured off. Then 0.5 mL of absolute ethanol was added into each tube, shaken evenly and centrifuged for another 15 min. The precipitate at the bottom was retained, but the liquid was poured off. The precipitate was air dried for 5 min, stored in the refrigerator at -80°C, and dissolved with 50 μL of DEPC-treated water in each tube.

### MRNA detection

The total RNA was extracted from the feces in the three groups using the Omega fecal RNA extraction kit (R6828-01), and one-step fluorescence qPCR was performed using kits (Biotend). The primer se qu ences were as follows: miR-21 F: 5’-TGGATATGGATGGTCA-GATGAA-3’, R: 5’-TTTCTTGCCGTTCTGTAAGTG-3’. TNF-α F: 5’-GCTGCACTTTGGAGTGATCG-3’, R: 5’-GAGGGTTTGCTACAACATGGG-3’. U6 F: 5’-CTCGC-TTCGGCAGCACA-3’, R: 5’-AACGCTTCACGAATT-TGCGT-3’. U6 was used as an internal reference for the experiment. The primers were syn thesized by Shanghai Biotend Biotechnology Co., Ltd. The relative gene expression level was evaluated and calculated using 2^-ΔΔCt^ method.

### Statistical analysis

Statistical Product and Service Solutions (SPSS) 22.0 software (IBM, Armonk, NY, USA) was used for analysis. The measurement data were expressed as mean ± standard deviation, and t-test was adopted for comparison between two groups, and F test for comparison among groups. The enumeration data were expressed as rate, and chi-square test was adopted for comparison. Linear correlation analysis was performed using Pearson correlation analysis. Risk factors for the seizure of UC were analyzed via multivariate logistic regression analysis. P<0.05 was considered to be statistically significant (^*#&^P<0.05).

## Results

### General clinical data of subjects

In this study, a total of 150 UC patients and 150 healthy controls were collected. According to gender matching in the two groups, there were 75 males and 75 females in the two groups. In UC patients, the average age was (57.2±9.2) years old, and BMI was (23.93±3.21) kg/m^2^. In control group, the average age was (56.3±8.9) years old, and BMI was (24.73±4.02) kg/m^2^. The gender, age and BMI had no statistically significant differences between the two groups (P>0.05) (Table 1). The above results indicate that the gender, age and BMI were balanced and comparable in the two groups.

**Table 1:** Comparison of regular indexes.

Variable	Control<br>(n=150)	UC<br>(n=150)	t/χ^2^	P
Sex<br>(male/female)	75/75	75/75	–	–
Age<br>(years old)	56.3±8.9	57.2±9.2	-0.861	0.390
BMI<br>(kg/m^2^)	24.73±4.02	23.93±3.21	1.905	0.058

### Comparison of intestinal flora among groups

Among the 150 UC patients, there were 82 cases in seizure group and 68 cases in remission group. According to the analysis of intestinal flora, the number of Bifidobacterium and Lactobacillus inseizure group ((5.31±0.44) lgCFU/g and (4.92±0.26) lgCFU/g) was obviously smaller than that in remission group and control group, and the differences were statistically significant (P<0.05). The number of Entero bacterium and Enterococcus in in seizure group ((12.44±1.03) lgCFU/g and (8.89±0.38) lgCFU/g) was obviously larger than that in remission group and control group, and the differences were statistically significant (P<0.05). Moreover, remission group had an evidently smaller number of Bifido bacterium and Lactobacillus (P<0.05) and an evidently larger number of Enterobacterium and Enterococcus than control group (P<0.05), showing statistically significant differences (P<0.05) (Table 2).

**Table 2:** Comparison of intestinal flora.

Variable	Control<br>(n=150)	Remission<br>(n=68)	Seizure<br>(n=82)	F	P
Bifidobacterium<br>(lg CFU/g)	10.37±0.65	8.57±0.54^#^	5.31±0.44^#&^	9.325	<0.001
Lactobacillus<br>(lg CFU/g)	9.56±0.84	7.55±0.63^#^	4.92±0.26^#&^	11.793	<0.001
Enterobacteriaceae<br>(lg CFU/g)	7.48±0.59	10.48±0.82^#^	12.44±1.03^#&^	8.972	<0.001
Enterococcus<br>(lg CFU/g)	7.07±0.24	8.11±0.34^#^	8.89±0.38^#&^	8.314	<0.001

### Comparison of miR-21 and TNF-α among groups

**Figure 1:**
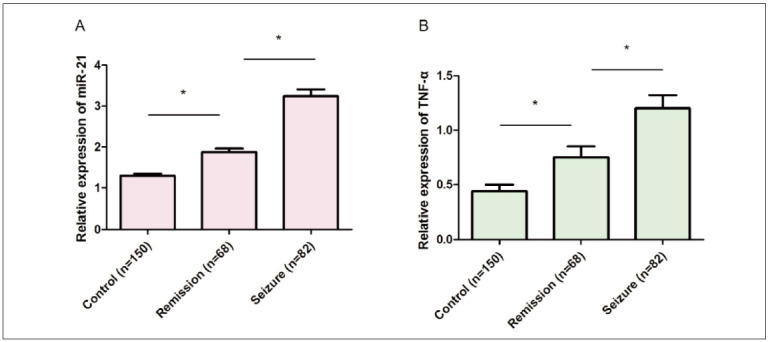
Comparison of miR-21 and TNF-α among groups. (A) The results of RT-PCR revealed that the expression of miR-21 was markedly higher in seizure group than that in remission group and control group, while it was also markedly higher in remission group than that in control group, and there were statistically significant differences (P<0.05). (B) The expression of TNF-α was markedly higher in seizure group than that in remission group and control group, while it was also markedly higher in remission group than that in control group, and there were statistically significant differences (P<0.05).

The results of RT-PCR revealed that the expression of fecal miR-21 was markedly higher in seizure group than that in remission group and control group, while it was also markedly higher in remission group than that in control group, and there were statistically significant differences (P<0.05) (Figure 1A). The expression of fecal TNF-α was markedly higher in seizure group than that in remission group and control group, while it was also markedly higher in remission group than that in control group, and there were statistically significant differences (P<0.05) (Figure 1B). These results indicate that the expressions of fecal miR-21 and TNF-α of UC patients are significantly up-regulated, more significantly with the aggravation of disease.

### Correlation between miR-21 and TNF-α

**Figure 2:**
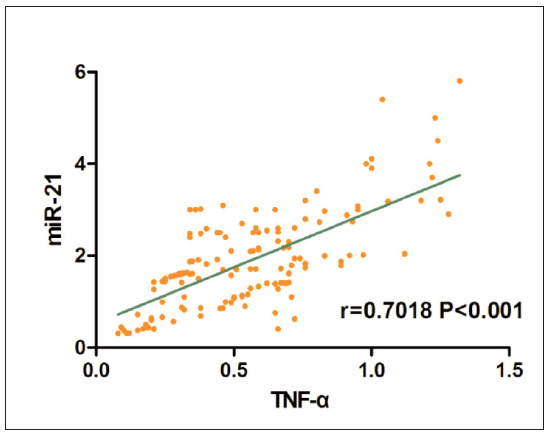
Correlations between miR-21 and TNF-α. The results of Pearson correlation analysis showed that there was a positive correlation between expressions of miR-21 and TNF-α (r=0.7018, P<0.001).

To explore whether there is a correlation between miR-21 and TNF-α, Pearson correlation analysis was conducted. It was found that there was a positive correlation between expressions of miR-21 and TNF-α (*r*=0.7018, P<0.001) (Figure 2), indicating that miR-21 can positively regulate the expression of TNF-α.

### Correlations of miR-21 and TNF-α with intestinal flora

To further explore whether miR-21 and TNF-α can affect the distribution of intestinal flora, Pearson correlation analysis was conducted again. It was found that Bifidobacterium and Lactobacillus werenegatively correlated with miR-21 (*r*=-0.596 & -0.368, P<0.05) and TNF-α (*r*=-0.682 & -0.427, P<0.05) in the three groups. Enterobacterium and Enterococcus were positively correlated with miR-21 (*r*=0.753 & 0.593, P<0.05) and TNF-α (*r*=0.727 & 0.641, P<0.05) in the three groups (Table 3). The above findings demonstrate that the expression levels of miR-21 and TNF-α will affect the distribution of intestinal flora.

**Table 3:** Correlation between miR-21/TNF-α expression and intestinal flora.

Variable	miRNA-21		TNF-α	
	r	P	r	P
Bifidobacterium<br>(lg CFU/g)	-0.596	0.007	-0.682	<0.001
Lactobacillus<br>(lg CFU/g)	-0.368	0.018	-0.427	0.006
Enterobacteriaceae<br>(lg CFU/g)	0.753	0.024	0.727	0.005
Enterococcus<br>(lg CFU/g)	0.593	0.011	0.641	0.034

### Multivariate logistic regression analysis of risk factors for UC

Multivariate logistic regression analysis was performed with UC as the dependent variable, and with intestinal flora (Bifidobacterium, Lactobacillus, Enterobacterium and Enterococcus), miR-21 and TNF-α as independent variables. The results showed that Bifido bacterium and Lactobacillus were protective factors against UC, while Enterobacterium, Enterococcus, miR-21 and TNF-α were risk factors for UC (P<0.05) (Table 4). According to the results of multivariate logistic regression analysis with the seizure of UC as the dependent variable, and with intestinal flora (Bifidobacterium, Lactobacillus, Enterobacterium and Enterococcus), miR-21 and TNF-α as independent variables, Bifidobacterium was the protective factor against the seizure of UC, while Lactobacillus, miR-21 and TNF-α were risk factors for the seizure of UC (P<0.05) (Table 5). It can be inferred that miR-21, TNF-α and alteration of intestinal flora will enhance the occurrence and severity of UC.

**Table 4:** Logistic regression analysis of risk factors for UC.

Variable	OR	95% CI	P
Bifidobacterium<br>(lg CFU/g)	0.587	0.249–0.772	0.016
Lactobacillus<br>(lg CFU/g)	0.791	0.462–0.813	0.003
Enterobacteriaceae<br>(lg CFU/g)	1.753	1.342–3.204	0.021
Enterococcus<br>(lg CFU/g)	1.347	1.291–1.873	0.008
miRNA-21	2.134	1.324–3.041	<0.001
TNF-α	1.795	1.242–2.824	0.004

**Table 5:** Logistic regression analysis of risk factors for UC seizure.

Variable	OR	95% CI	P
Bifidobacterium<br>(lg CFU/g)	0.772	0.583–0.872	0.007
Lactobacillus<br>(lg CFU/g)	0.823	0.649–1.724	0.081
Enterobacteriaceae<br>(lg CFU/g)	1.696	1.187–1.753	<0.001
Enterococcus<br>(lg CFU/g)	1.294	0.973–3.237	0.175
miRNA-21	1.876	1.542–2.341	0.001
TNF-α	1.642	1.324–1.863	<0.001

## Discussion

A multitude of microorganisms adhere to the human intestine, playing crucial roles in maintaining normal physiological functions and stabilizing the immune system of the host. The intestinal flora is a significant factor in the development of the host’s immune system [14] [15], and in turn, the host’s immune system affects the structure and function of the intestinal flora [16]. An alteration in the intestinal flora can lead to the occurrence and progression of several digestive tract diseases. One study indicated that the interaction between the body and the intestinal flora should be considered as a part of the body when diagnosing and treating diseases [17].

The intestinal flora is part of a more intricate micro-ecosystem. Beneficial bacteria such as Bifidobacterium and Lactobacillus produce bacteriocins and organic acids that resist the invasion of conditional pathogenic bacteria into the human intestinal mucosa. In the case of an imbalance in the intestinal flora, conditional pathogenic bacteria like Enterobacterium and Enterococcus may have enhanced invasiveness, thereby damaging the intestinal mucosa of the body [18] [19].

Related research suggests that miR-21 can hinder the apoptosis of activated T cells, and its overexpression may promote T cell-mediated inflammatory response by regulating abnormal T cell response. It may also impact T helper cells and cause functional defects through interleukin-2 and B-cell lymphoma-2, which could be a significant pathogenic factor in UC [13] [20]. Sheedy et al. [21] demonstrated that miR-21 negatively regulates the TLR4 pathway through PDCD4, and its expression increases in the intestinal mucosa of patients with active UC, indicating that miR-21 may be involved in the pathogenesis of UC. TNF-α, an essential pro-inflammatory cytokine, cansynergize with various cytokines to further induce the release of inflammatory mediators in the body [22]. Moreover, TNF-α is regulated by miR-21 and has a crucial role in the occurrence of acute myocardial infarction in the elderly [12]. In this study, the results revealed that the upregulation of miR-21 and TNF-α was closely associated with the occurrence and development of UC. The expressions of miR-21 and TNF-α could reflect the severity of UC to a certain extent and were positively correlated with each other.

Considering the close relationship between changes in intestinal flora and miR-21 in UC, alterationsin the intestinal flora were evaluated by measuring the levels of Bifidobacterium, Lactobacillus, Entero bacterium, and Enterococcus. Correlations between these levels and miR-21 and TNF-α were then analyzed to provide important insights into the pathogenesis of UC. The results of the Pearson correlation analysis showed that miR-21 and TNF-α were negatively correlated with the content of Bifidobacterium and Lactobacillus, but positively correlated with the content of Enterobacterium and Enterococcus, confirming that changes in the intestinal flora are associated with changes in the levels of miR-21 and TNF-α. Moreover, the results of multivariate logistic regression analysis indicated that Bifidobacterium and Lactobacillus are protective factors against UC, while Enterobacterium, Enterococcus, miR-21, and TNF-α are risk factors for UC. Additionally, Bifidobacterium was found to delay the progression of UC, while Enterobacterium, miR-21, and TNF-α were found to promote the development of UC.

In summary, individuals with UC exhibit higher levels of miR-21 and TNF-α in their feces than healthy individuals, and are susceptible to changes in their intestinal flora. Alterations in intestinal flora, miR-21, and TNF-α may all play important roles in the pathogenesis and progression of UC. Additionally, measuring the levels of miR-21 and TNF-α may aid in the clinical evaluation of disease severity.

## Conclusion

MiR-21 can promote the expression of TNF-α, and lead to the alteration of intestinal flora, thereby enhancing the occurrence and development of UC.

## Dodatak

### Conflict of interest statement

All the authors declare that they have no conflict of interest in this work.
